# Controlled delivery of BID protein fused with TAT peptide sensitizes cancer cells to apoptosis

**DOI:** 10.1186/1471-2407-14-771

**Published:** 2014-10-18

**Authors:** Emilia Joanna Orzechowska, Ewa Kozlowska, Alicja Czubaty, Piotr Kozlowski, Krzysztof Staron, Joanna Trzcinska-Danielewicz

**Affiliations:** Department of Molecular Biology, Faculty of Biology, University of Warsaw, Miecznikowa 1, 02-096 Warsaw, Poland; Department of Immunology, Faculty of Biology, University of Warsaw, Miecznikowa 1, 02-096 Warsaw, Poland

**Keywords:** Apoptosis, BID, Camptothecin, Cancer cells, TAT peptide, TRAIL

## Abstract

**Background:**

Low cellular level of BID is critical for viability of numerous cancer cells. Sensitization of cells to anticancer agents by BID overexpression from adenovirus or pcDNA vectors is a proposed strategy for cancer therapy; however it does not provide any stringent control of cellular level of BID. The aim of this work was to examine whether a fusion of BID with TAT cell penetrating peptide (TAT-BID) may be used for controlled sensitization of cancer cells to anticancer agents acting through death receptors (TRAIL) or DNA damage (camptothecin). Prostate cancer PC3 and LNCaP, non-small human lung cancer A549, and cervix carcinoma HeLa cells were used in the study.

**Methods:**

Uptake of TAT-BID protein by cells was studied by quantitative Western blot analysis of cells extracts. Cells viability was monitored by MTT test. Apoptosis was detected by flow cytometry and cytochrome c release assay.

**Results:**

TAT-BID was delivered to all cancer cells in amounts depending on time, dose and the cell line. Recombinant BID sensitized PC3 cells to TRAIL or, to lesser extent, to camptothecin. Out of remaining cells, TAT-BID sensitized A549, and only slightly HeLa cells to TRAIL. None of the latter cell lines were sensitized to camptothecin. In all cases the mutant not phosphorylable by CK2 (TAT-BID^T59AS76A^) was similarly efficient in sensitization as the wild type TAT-BID.

**Conclusions:**

TAT-BID may be delivered to cancer cells in controlled manner and efficiently sensitizes PC3 and A549 cells to TRAIL. Therefore, it may be considered as a potential therapeutic agent that enhances the efficacy of TRAIL for the treatment of prostate and non-small human lung cancer.

**Electronic supplementary material:**

The online version of this article (doi:10.1186/1471-2407-14-771) contains supplementary material, which is available to authorized users.

## Background

Susceptibility to apoptosis is impaired in majority of cancer cells. A common reason is reduction of the "functional dose" of apoptotic proteins [1], i.e. decrease of effective concentration of active proteins below a critical level necessary to carry out the process. The main causes of the reduction are: (i) lowered expression of apoptotic proteins, e.g. caspases [2]; (ii) elevated expression of inhibitors of apoptosis proteins (IAPs) that reduce the level of the initiator and effector caspases [3], and (iii) phosphorylation of apoptotic proteins [4, 5] that makes them inaccessible to signaling and eventually prevents apoptosis in cancer cells. Reconstruction of the physiological functional dose of apoptotic protein in protein-deficient cells is a suggested strategy for cancer therapy.

BID (**B**H3-**i**nteracting domain **d**eath agonist) protein is situated in extrinsic apoptotic signaling between death receptors and mitochondria [6], and acts as an inductor of permeabilization of the outer mitochondrial membrane in type II cells [7]. The level of BID is critical for viability of numerous cells because its silencing makes them resistant to apoptosis induced by death receptors ligands, e.g. TNF-related apoptosis-inducing ligand (TRAIL) [8, 9]. Moreover, it has been demonstrated that the level of BID is below the functional dose in cells of several lines because they may be sensitized to TRAIL by overexpression of BID [8, 10, 11]. Due to the above, BID has been considered to be therapeutically exploited [8]. However, to define a way to administer BID several significant issues should be solved. The main one is a control of the level of BID delivered to the cell. Although a full-length BID has been shown to participate in apoptotic signaling [12], efficient activation of apoptosis needs a specific cleavage of BID by caspase 8 and production of an active truncated form (tBID) [13]. tBID expressed in cells directly induces apoptosis [10]. Therefore, to exploit selectivity for cancer cells exhibited by some anticancer agents [14, 15] sensitization of cells by full-length BID is a preferred strategy. To sensitize cells, overexpressing systems based on the adenovirus [11] or pcDNA vectors [8, 16, 17] have been commonly used. However, they do not provide a stringent control of the level of BID expression in the cell. As a result, the level of BID in transfected cells exceeded several fold that of the endogenous protein and in some cases a direct activation of apoptosis was observed instead of sensitization of cells to apoptotic stimuli [10, 11, 16]. In according to the above, tBID appears in cells treated with adenovirus vector expressing the full-length BID [11, 16].

Another question to be answered is whether there is a critical level of endogenous BID that would be prediction for BID-based therapy. Estimated levels of endogenous BID vary from one cell type to another by a factor of about one hundred [18]. However, efficient use of cellular BID may be hampered due to at least two reasons. Firstly, active tBID is sequestered by Bcl-2 proteins [19]. As a consequence, higher level of BID is necessary to support signaling in cells overexpressing Bcl-2. Next, the level of available BID may be reduced by phosphorylation of the protein by CK2 kinase. The cleavage of BID by caspase 8 that produces tBID has been suggested to be dependent on phosphorylation status of BID regulated by CK2 [5, 20]. Although BID is a poor substrate for CK2 [21], phosphorylation of BID by CK2 in HeLa cells protects it partially from cleavage by caspase 8 [22]. Therefore, as the level of endogenous BID [18], functioning of TRAIL-induced signaling [23], and activity of CK2 [24] differ in particular cancer cells, some cells may be expected not to respond on elevation of cellular concentration of BID whereas other ones may be sensitized by BID.

An issue to be clarified is also which apoptosis inducing agents could be combined with BID to achieve an anticancer effect. Due to the role of BID in the extrinsic apoptotic signaling sensitization with BID is usually followed by a treatment of cells with death receptors ligands [8, 9]. From among them soluble human TRAIL (Dulanermin) is considered as a potential anticancer drug. It exhibits cancer-selective effects [14, 15]. Clinical evidence shows that TRAIL is relatively safe and well tolerated [25], and several treatment strategies based on recombinant soluble TRAIL have been recently built and tested ( [26] for review). Attempts have been undertaken to improve delivery of TRAIL to target cells and its therapeutic efficacy [15]. Another cellular role proposed for BID is to mediate DNA damage response [27]. It has been shown that BID promotes intrinsic apoptotic pathway in hepatocellular carcinoma cells (HCC) because they are sensitized to etoposide by overexpression of BID [17, 28]. However, it is not clear whether sensitization of the intrinsic apoptotic pathway by BID is a general phenomenon or it is limited to HCC, and which DNA damaging agents would be a therapeutic option, if they were combined with BID.

With regard to the potential use of BID as a target in cancer therapy, we addressed here the issues listed above by employing TAT cell penetrating peptide to deliver recombinant BID to cancer cells. TAT peptide is one of the smallest protein-transduction domains [29] considered as a vehicle to deliver proteins into cancer cells in anti-cancer therapies [30]. It was exploited to transport several different apoptotic proteins either individually e.g. [31, 32] or as a fusion with domains that specifically confine their action to cancer cells e.g. [33, 34]. Prostate cancer PC3 cells were the main subject of this study. They have been isolated from bone metastasis of prostate cancer and retain high metastatic potential. PC3 cells are commonly used as a model of prostate cancer metastasis e.g. [35, 36], poorly susceptible to chemotherapy. They are type II cells [37] that use an apoptotic pathway dependent on the presence of BID [38]. CK2 activity in prostate cancer cells is ~3-5 times higher than in a normal tissue [39] and it influences susceptibility of these cells to apoptosis [40]. Moreover, impairment of apoptosis and not increased proliferation is a main factor in prostate cancer progression [41] making it a promising target for BID-based therapy. To have a better insight into ability of directly delivered BID to sensitize different cancer cells, we also tested three other cancer cell lines: prostate cancer LNCaP, non-small human lung cancer A549 and cervix carcinoma HeLa. TRAIL and camptothecin (CPT) were used to induce apoptosis through either death receptors or DNA damage, respectively. We found that: (i) BID fused with TAT peptide is delivered to cells at the non-toxic dose; (ii) delivered BID sensitized PC3 cells to apoptosis induced by TRAIL and CPT; (iii) out of other cancer cell lines A549 and HeLa cells were sensitized to apoptosis induced by TRAIL and no line to apoptosis induced by CPT; (iv) unphosphorylable mutant of BID sensitized cells similarly as the wild type protein.

## Methods

### Cell lines and cell culture

Experiments were performed using two prostate cancer cell lines (PC3 and LNCaP), and two non-prostate cell lines: non-small human lung cancer A549 and cervix carcinoma HeLa. All cell lines were purchased from the European Collection of Cell Cultures (ECACC). Prostate cancer cells were cultured in RPMI-1640 medium. PC3 medium contained 2 mg/ml D-glucose. LNCaP medium contained 4.5 mg/ml D-glucose, and additionally 10 mM HEPES and 1 mM sodium pyruvate. Non-small human lung cancer A549 cell line and cervix carcinoma HeLa cell line were cultured in F12K or DMEM medium, respectively. All media were supplemented with 10% FBS, penicillin (100 U/ml) and streptomycin (100 μg/ml). Cultures were maintained in a 95% humidified atmosphere of 5% CO_2_ at 37°C. For experiments cells were seeded in 96- and 24-wells plates or dishes.

### Reagents

*Pfx* polymerase was obtained from Invitrogen (Thermo Fisher Scientific, USA); Ni-NTA agarose resin, GAPDH antibodies and RPMI-1640 medium from Sigma ALDRICH (Inc. Sigma-Aldrich Corp, MO, USA); F-12 K medium from ATCC (ATTC, VA, USA); Applixchange-G25M from AppliChem (AppliChem GmbH, Darmstadt, Germany); Superdex-200 from Amersham (GE Healthcare Europe GmbH, Austria); anti-Bid antibodies from Santa Cruz Biotechnology, Inc. (Santa Cruz, CA, USA); cytochrome c antibodies and Annexin V-FITC Apoptosis Detection kit I from Becton and Dickinson Bioscience (Becton, Dickinson and Company, New Jersey, USA); Protease Inhibitor Cocktail from Promega (Promega Corporation, USA).

### Plasmid construction and mutagenesis

cDNA corresponding to human BID (BID(L), isoform 1, 195 aa) was amplified by PCR method. Plasmid IRATp970C1135D (imaGenes) containing full length cDNA BID clone [GenBank: BC036364] was used as a template. To construct pET28a/TAT-BID plasmids encoding a series of BID fusion proteins, bacterial vector pET28a (Novagen) was enriched with sequences coding TAT and repeated three times HA-tag. BID cDNA was cloned into the vector between TAT-sequence and HA-tags. All mutations were introduced into constructed plasmid by site-directed mutagenesis.

Plasmid for expression of human soluble TRAIL was prepared as described previously [42].

### Expression, isolation and purification of recombinant proteins

**Figure 1:**
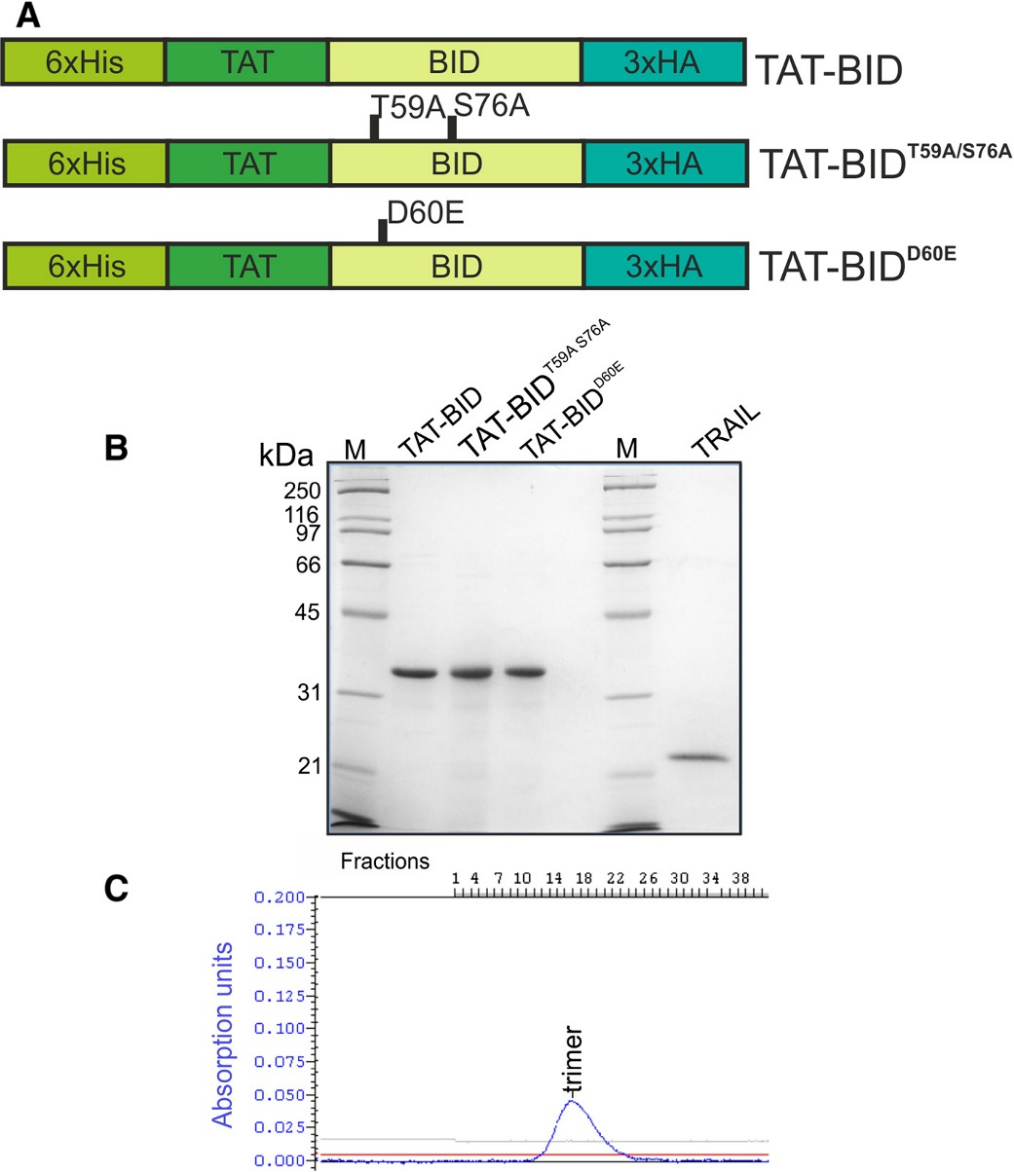
**Recombinant proteins used in this work. A**. Schematic representation of TAT-BID constructs used in this study. **B**. Purity of recombinant TAT-BID, its mutants, and TRAIL. The gel was stained with Coomassie Brilliant Blue R-250. **C**. Chromatography of TRAIL on Superdex 200 (original picture is shown; straight lines at the bottom illustrate controls). Position of the trimeric form of TRAIL is marked.

Recombinant BID protein fused with TAT peptide was used in this study (Figure 1A). The construct was used either as a wild type protein (TAT-BID) or its mutated variants. In the latter case, the fusion protein mutated at sites phosphorylated by CK2 kinase [21], (TAT-BID^T59A/S76A^) was used for testing sensitivity of exogenous BID to phosphorylation by CK2 in cancer cells, and the mutant uncleavable by caspase 8 [43] (TAT-BID^D60E^) for testing the processing of delivered recombinant BID in the cell. All the above mentioned proteins were tagged with His-tag used for purification and with HA tag used for simple identification of the protein in the cell. His-tag used for purification and the TAT peptide used for the cell penetration were localized at the N-terminal end of the protein and thus they were removed after cleavage by caspase 8 that makes the protein active. On the other hand, HA tags used for identification of the protein were placed at the C-terminus and remained uncut after proteolytic cleavage.

Recombinant BID was expressed in *Escherichia coli* BL21(DE3) cells. The proteins were isolated and purified under native conditions using Ni-NTA agarose resin and gel-filtration (Applixchange-G25M) chromatography. Purity of all variants of recombinant TAT-BID is shown on Figure 1B. Recombinant soluble form of human TRAIL [14] was used to induce apoptosis in several experiments. For TRAIL protein expression *E. coli* M15 [pREP4] (Qiagen) cells were used. TRAIL was purified under native conditions using Ni-NTA agarose resin and then using FPLC on Superdex-200. Purity of TRAIL is shown on Figure 1B. Estimation of the molecular weight identified that nearly 100% of prepared TRAIL was in an active trimeric form [14] (Figure 1C).

Protein concentration in samples used in the experiments was quantified by densitometry after SDS-polyacrylamide gel electrophoresis using ChemiDocXRS (BioRad).

### Uptake of the recombinant proteins by cells

To examine time-dependent kinetics and concentration-dependence of uptake of the recombinant protein by the cells, TAT-BID was added directly to the culture medium in the presence of trypsin inhibitor from soybean (final concentration 0.005%). Then cells were washed 3 times with PBS, trypsinized, collected and centrifuged at 250 × g for 5 min at 4°C. 3 additional series of washing were made and then cells were counted, resuspended in Laemmli buffer (1×10^4^ cells/μl) and boiled for 20 min, resolved on a polyacrylamide gel under denaturing conditions, and transferred into PVDF membrane. Protein concentration in extracts was measured using Bradford assay [44]. Protein’s uptake and the level of endogenous BID were detected by Western blot analysis carried out with the aid of anti-Bid antibodies. The membranes were subsequently stripped of the first antibody and re-probed with anti-GAPDH antibodies. The results were quantified by densitometry using ChemiDocXRS (BioRad).

### MTT assay and detection of apoptosis

MTT mixture [45] was added at the concentration 0.5 mg/ml for PC3, LNCaP and A549 cells or 2.5 mg/ml for HeLa cells, after removal of the culture medium. Cells were then cultured for additional 120 min at 37°C. The formazan crystals were diluted in DMSO–isopropanol mixture (1:1) and the absorbance was measured at 570 nm using VICTOR Multilabel Plate Reader (PerkinElmer). For detection of apoptosis PC3 cells were cultured for 24 hours with 30 μg/ml of TAT-BID in the presence of trypsin inhibitor from soybean and 200 ng/ml TRAIL. Then, both floating and adherent cells were harvested. The floating cells were collected by centrifugation at 700 × g for 5 min at 4°C. Adherent cells were first trypsinized and then collected by centrifugation at 700 × g for 5 min at 4°C. Both fractions were resuspended in Annexin V binding buffer and pooled. Apoptotic cells were detected by flow cytometry as described previously [42] and analyzed with a BD FACSDiva Software ver. 6.0 (Becton Dickinson).

### Cytochrome c assay

To detect cytochrome c release, cytosol-free mitochondria were prepared. PC3 cells were seeded on 60 mm dishes and treated with TAT-BID (30 μg/ml), TRAIL (100 ng/ml) or TAT-BID + TRAIL for 4 hours. Next, both floating and adherent cells were collected, spun at 1000 g for 5 min at RT and pellets were resuspended in ice-cold Mito-buffer (20 mM HEPES, pH 7.5, 10 mM KCl, 1.5 mM MgCl_2_, 1 mM EGTA, 1 mM EDTA, 1 mM DTT, 250 mM sucrose, 100 μM benzamidine, 55 μM phenanthroline, 20 μM leupeptin, 5 μM pepstatin A, 1 mM PMSF) and lysed on ice by homogenization in a small glass Potter-Elvehjem homogenizer. The homogenates were spun at 800 g for 10 min at 4°C to remove nuclei and cell detritus and then spun at 16 000 g for 30 min at 4°C to pellet the mitochondria. Pellets were washed with Mito-buffer and spun again at 16 000 g for 30 min at 4°C [46]. Cytosol-free mitochondria were lysed in Laemmli buffer, boiled for 10 min at 95°C and used for Western blot analysis. Mitochondrial proteins were separated on 15% polyacrylamide gel and cytochrome c was detected using anti-cytochrome c antibodies. Also GAPDH protein was detected by Western blot analysis using anti-GAPDH antibodies. The results were quantified by densitometry using ChemiDocXRS (BioRad).

### Data analysis

All experiments were carried out at least in triplicate, and for each individual point at least five independent measurements were done. Differences between groups were calculated using t-Student test. P-Value < 0.05 was considered statistically significant. The following ranges were defined: p < 0.05 (*), p < 0.01 (**) and p < 0.001 (***). Statistica ver10 software (StatSoft, Poland) was used for analysis.

## Results

### TAT-BID enters PC3 cells in amounts depending on time and dose

**Figure 2:**
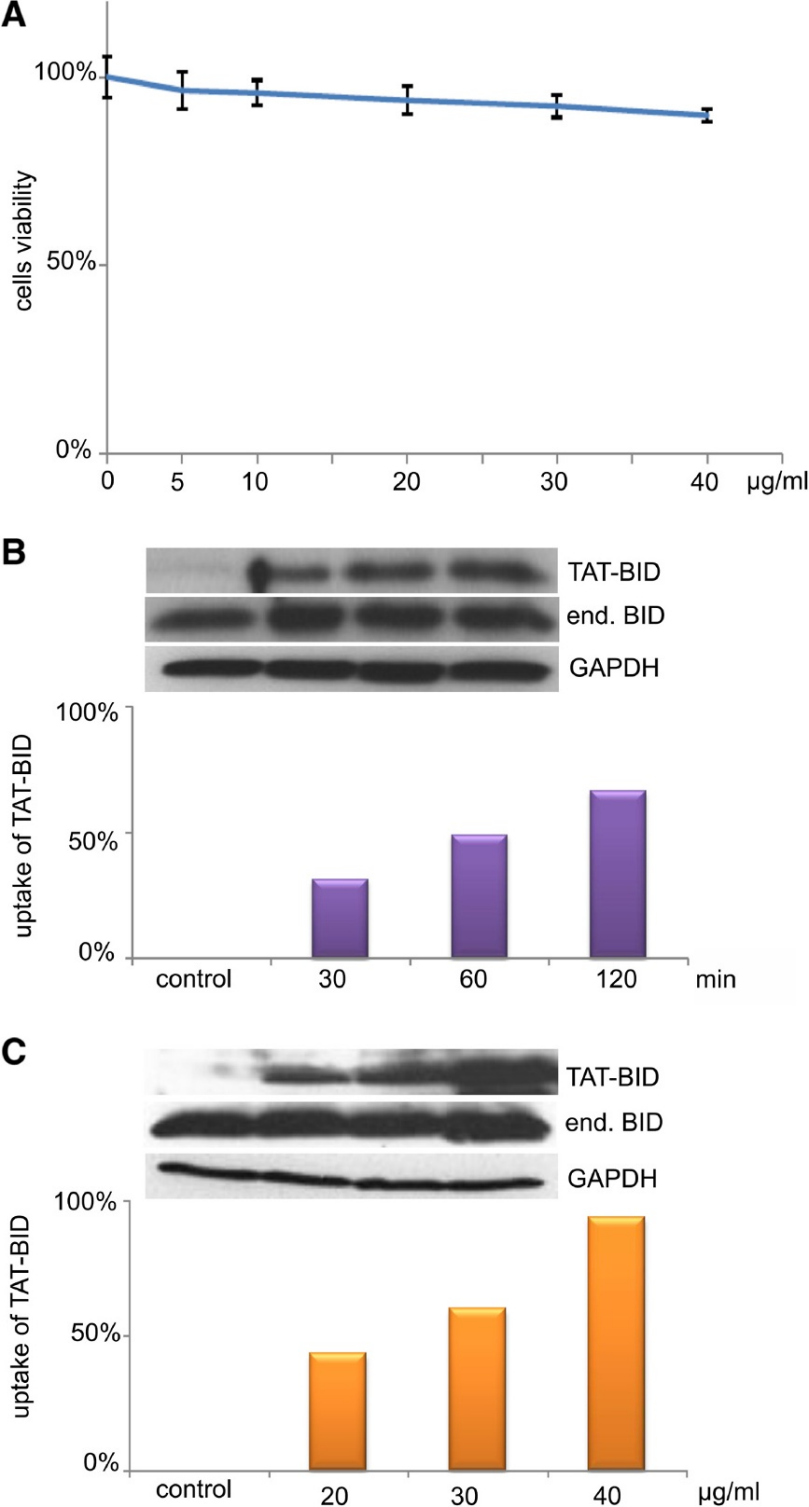
**Uptake of TAT-BID by PC3 cells. A**. Effect of different doses of TAT-BID on PC3 cells viability. Viability of cells treated with TAT-BID for 24 h was measured by MTT test. The average of cells viability (±SD) was shown. **B**. Time-dependence of the uptake of TAT-BID by PC3 cells. TAT-BID was added to the medium at a concentration of 30 μg/ml. **C**. Dose-dependence of TAT-BID uptake by PC3 cells. Different doses of TAT-BID were added to the medium for 120 min. TAT-BID and endogenous BID were identified in cell extracts using anti-BID antibodies. GAPDH was used as a loading control. Uptake was expressed as increase in cellular BID (endogenous level of BID was 100%).

To exert an effect on apoptosis TAT-BID had to be efficiently taken up by PC3 cells at a non-toxic dose. Therefore, we firstly examined whether TAT-BID entered PC3 cells, and whether a meaningful amount of recombinant BID appeared in the cells under conditions that not significantly influences their viability. Relatively short drug treatment is necessary for activation of early stages of apoptosis by TRAIL [47] and CPT [48]. We observed this phenomenon also in our experimental system where treatment with TRAIL for 1 h or with CPT for 2 h followed by incubation in a TRAIL- and CPT-free medium was enough to induce apoptosis detected after 24 h in all tested cancer cell lines (not shown). Therefore, to control increase in BID level, we examined the amount of TAT-BID taken up by the cells during the first two hours. We found that TAT-BID alone did not influence viability of PC3 cells by more than 10% within the range of concentrations used in this work, i.e. up to 40 μg/ml (Figure 2A). Therefore, we examined a dose dependence of transportation of TAT-BID within the range of 0 – 40 μg/ml.TAT-BID was taken up by PC3 cells in a time-dependent (Figure 2B) and a dose-dependent manner (Figure 2C). When the amount of TAT-BID was related to the level of endogenous BID, an amount of cellular BID increased under standard conditions of the treatment (30 μg/ml for 120 min) by about 65%. Taking into account the estimated level of endogenous BID in PC3 cells as 16.0 ± 4.4 fg/cell, the final level of cellular BID increased due to the treatment up to about 25 fg/cell. Both mutated TAT-BID were taken up in a similar extent as the wild type protein.

### TAT-BID sensitizes PC3 cells to apoptosis

**Figure 3:**
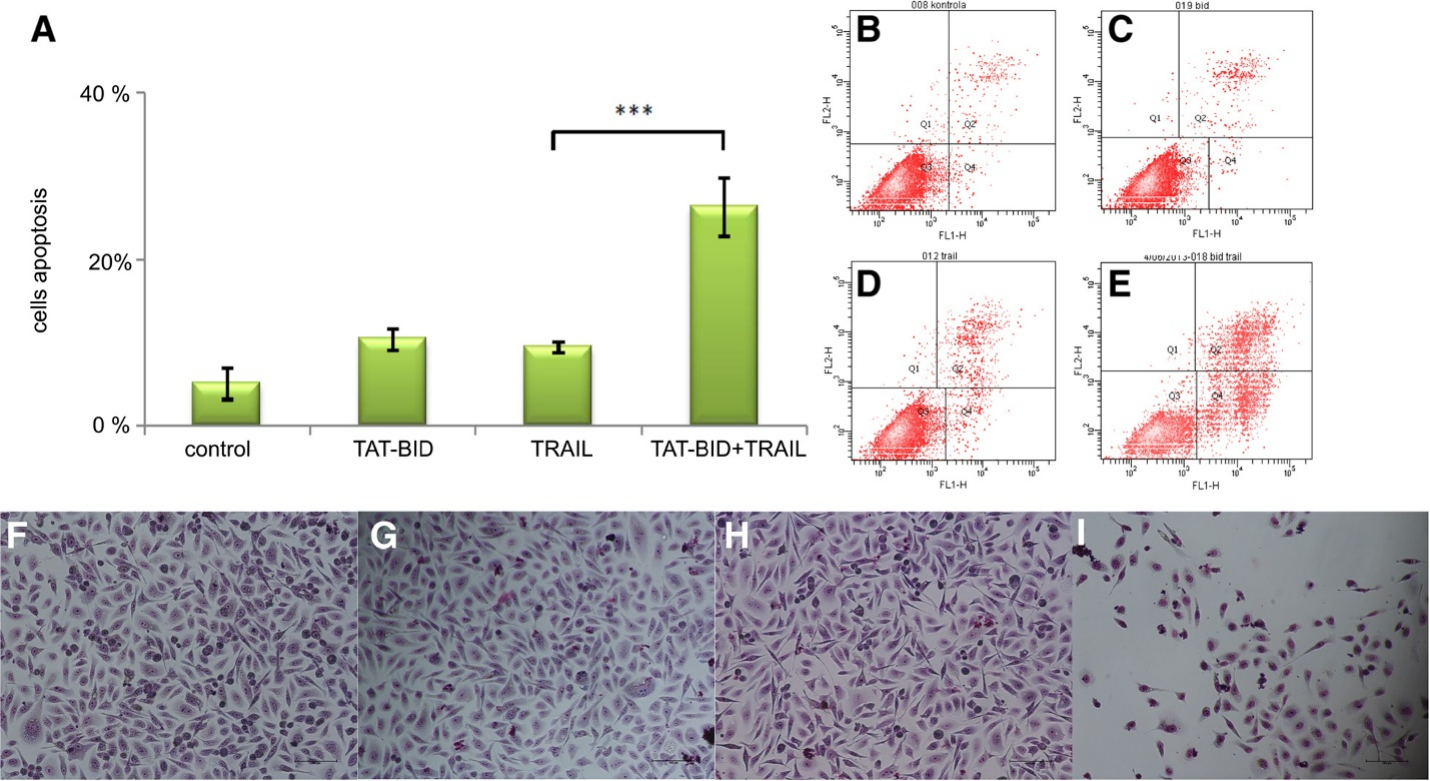
**Effect of TAT-BID on apoptosis induced in PC3 cells by TRAIL.** PC3 cells were incubated with TAT-BID (30 μg/ml) and TRAIL (200 ng/ml) for 24 h. **A**. Percentage of apoptotic cells detected by flow cytometry. The average of number of apoptotic cells (±SD) was shown. **B-E**. Representative cytograms. **F-I**. Microscopic pictures of the cells. Cells were stained with Giemsa and May-Grünwald dyes. **B**, **F**. Control cells. **C**, **G**. Cells treated with TAT-BID. **D**, **H**. Cells treated with TRAIL. **E**, **I**. Cells treated with TAT-BID and TRAIL.

**Figure 4:**
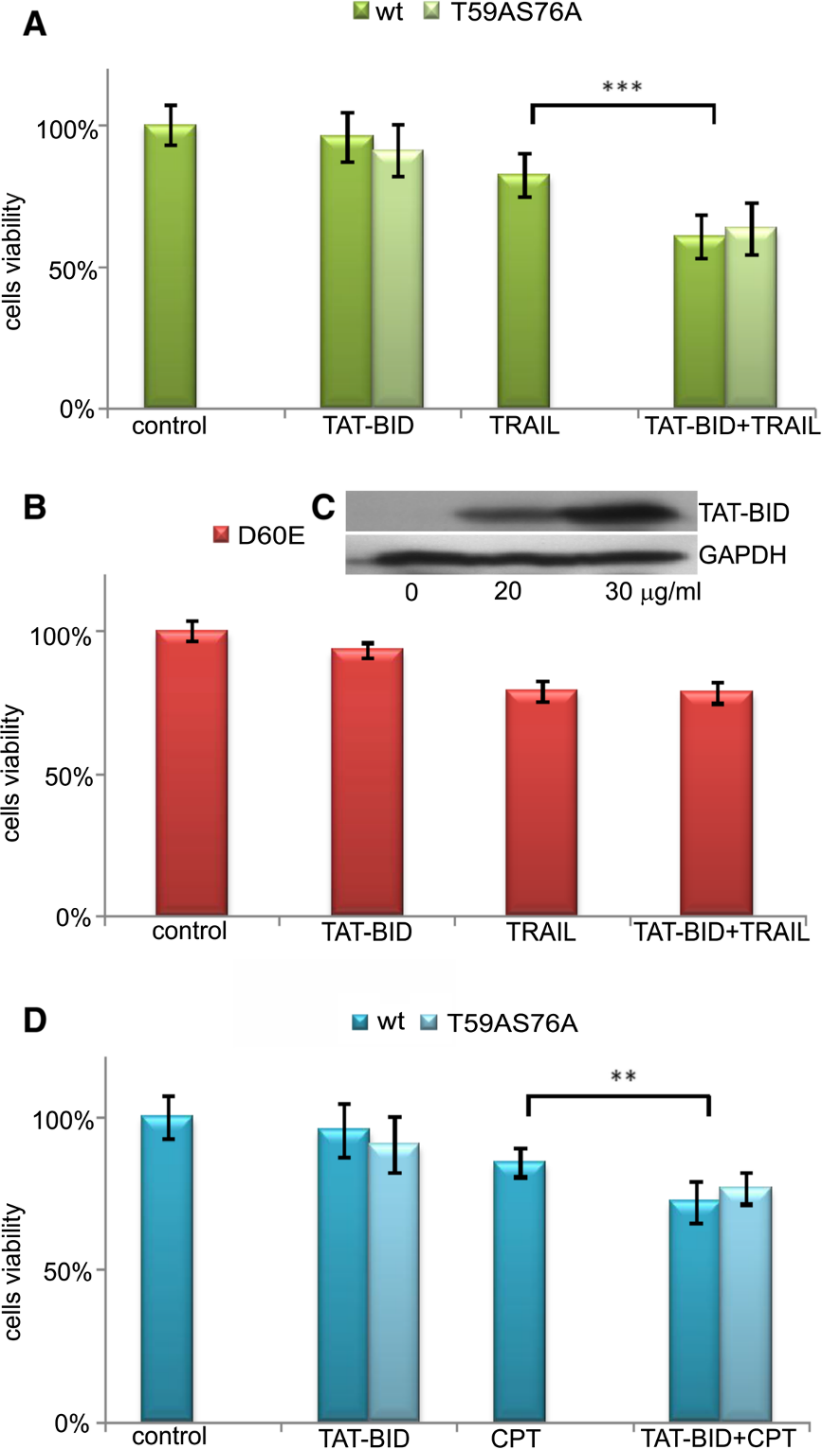
**Effect of TAT-BID on viability of PC3 cells treated with TRAIL or CPT. A**. Viability of cells treated with 30 μg/ml of the wild type TAT-BID (light bars) or TAT-BID^T59AS76A^ (dark bars) and 100 ng/ml of TRAIL for 24 h. **B**. Viability of cells treated with 30 μg/ml of TAT-BID^D60E^ and 200 ng/ml of TRAIL for 24 h. There is no statistically significant difference between viability of TRAIL- and TAT-BID^D60E^-treated cells. **C**. Dose-dependent TAT-BID^D60E^ uptake by PC3 cells measured after 120 min. **D**. Viability of cells treated with 30 μg/ml of TAT-BID and 30 nM of CPT. Viability of cells treated with TAT-BID for 24 h was measured by MTT test. The average of cells viability (±SD) was shown. **A** and **D**. Viability of control (control) and TRAIL- or CPT-treated cells (TRAIL or CPT, respectively) are valid in each case for both TAT-BID and TAT-BID^T59AS76A^.

We asked next whether increase of the cellular level of BID achieved due to controlled delivery of the protein resulted in increased sensitivity of PC3 cells to apoptosis induced by TRAIL. We found that TAT-BID increased a number of apoptotic cells that appeared after TRAIL treatment (Figure 3A-E). Such a picture was observed at a TRAIL dose that alone increased a number of apoptotic cells only by about 5 - 10% percent (Figure 3A; see also Additional file 1: Figure S1A). TAT-BID elevated this number by about 15 – 20 percentage points (Figure 3A) and this increment was relatively stable for higher concentrations of TRAIL (not shown). If calculated number of apoptotic cells was reduced by number of these cells present in the culture under control conditions at low concentrations of TRAIL, sensitization of PC3 cells by TAT-BID resulted in two- threefold increase of apoptosis as compared to cells treated with TRAIL alone (Figure 3A). Pro-apoptotic effect of combined treatment with TAT-BID and TRAIL can be observed on microscope pictures (Figure 3F-I). Increase of the amount of apoptotic cells upon combined TAT-BID + TRAIL-treatment of PC3 cells, as compared to cells treated with TRAIL alone, was also observed as a decrease in viability of the cells (MTT test) (Figure 4A).

Cytotoxicity of TAT peptide has been repeatedly examined and it was found to be low e.g. [49]. However, we would like to know whether the effects described above resulted from participation of recombinant BID in apoptosis and not from any unspecific action of TAT-BID fusion. To this end we used TAT fused with D60E mutant of TAT-BID that is not cleaved by caspase 8 [43]. We observed that TAT fused with inactive BID (TAT-BID^D60E^) was ineffective in sensitizing PC3 cells to TRAIL (Figure 4B), in spite of the fact that it entered into the cells (Figure 4C) in amounts similar to those of active protein. This indicates that cleavage of BID by caspase 8 [13] is a necessary prerequisite for effective action of TAT-BID.

The unphosphorylable mutant TAT-BID^T59AS76A^ was similarly effective as the wild type TAT-BID in sensitization of PC3 cells to TRAIL (Figure 4A). This indicates that phosphorylation of BID by CK2 suggested to prevent BID from immediate cleavage by caspase 8 [22] is not a limiting factor for efficacy of TAT-BID in PC3 cells.

BID is active not only in apoptotic signaling between death receptors and mitochondria [6, 13] but it also plays a role in the DNA damage response [17, 27]. Therefore, we asked next whether TAT-BID supported the latter apoptotic pathway in PC3 cells. To examine the effect of delivery recombinant BID on apoptosis induced by DNA damage we used CPT. The cells were sensitized by TAT-BID to CPT at the statistically significant level (Figure 4D); however, the effect was lower for CPT than for TRAIL used to induce apoptosis (compare Figures 4A and D). The mutant TAT-BID^T59A/S76A^ was similarly effective as the wild type TAT-BID in sensitization of PC3 cells to CPT.

### TAT-BID improves activation of the mitochondrial pathway in PC3 cells

**Figure 5:**
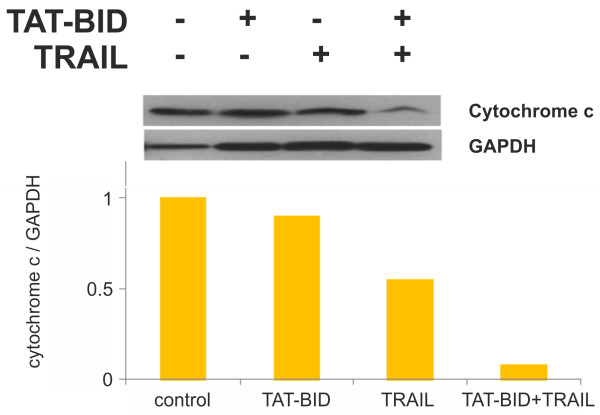
**Effect of TAT-BID on cytochrome c release from mitochondria of PC3 cells treated with TRAIL.** Cells were treated with TAT-BID or TRAIL (or both) for 4 h. Cytochrome c and GAPDH were identified in mitochondrial extracts using specific antibodies.

We also liked to know whether delivered TAT-BID was used in the cells to improve the mitochondrial cell death pathway. A critical event in the mitochondrial pathway is cytochrome c release [6]. Thus, we examined the effect of TAT-BID on TRAIL-induced cytochrome c release. We observed significant decrease of the amount of cytochrome c in mitochondrial fraction after combined TAT-BID + TRAIL treatment as compared to TAT-BID or TRAIL administered alone (Figure 5). Glyceraldehyde-3-phosphate dehydrogenase (GAPDH), commonly used as a load control because of its stable level, has been reported to accumulate in mitochondria of some cancer cell lines upon induction of apoptosis, and to participate in release of pro-apoptotic proteins from mitochondria [50]. If such a process occurred in PC3 cells, it was not detected by applied methods. However, regardless of whether it occurred or not, the cytochrome c/GAPDH ratio well illustrates activation of mitochondrial cell death pathway because both release of cytochrome c and putative accumulation of GAPDH are thought to participate in the pathway, and both alter cytochrome c/GAPDH ratio in the same direction. As it can be seen on Figure 5, the ratio was only slightly affected by TAT-BID, more seriously by TRAIL acting alone, and it dramatically dropped when TAT-BID was administered together with TRAIL.

### TAT-BID selectively sensitizes other cell lines to TRAIL

**Figure 6:**
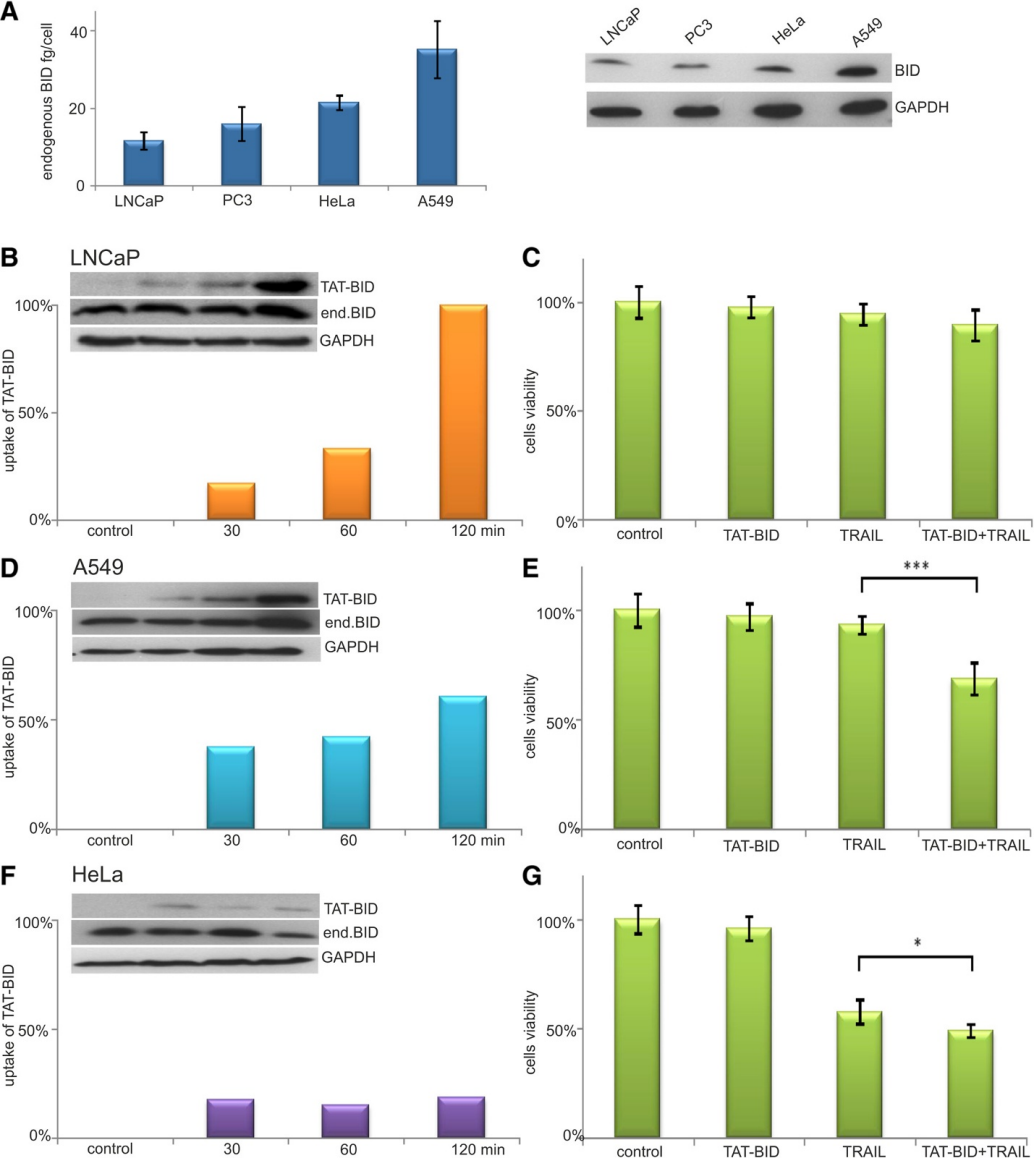
**Effect of TAT-BID on viability of different cancer cell lines treated with TRAIL. A**. Levels of endogenous BID in the studied cells. Endogenous BID was identified in cell extracts using anti-BID antibodies. GAPDH was used as a loading control. **B**, **D**, **F** – Time-dependence of the uptake of TAT-BID by the cells expressed as increase of cellular BID level (endogenous level of BID is 100%). Cells were treated with either 30 μg/ml (LNCaP) or 40 μg/ml of TAT-BID (A549 and HeLa) for 120 min. BID was identified in the cell extracts using anti-BID antibodies. GAPDH was used as a loading control. **C**, **E**, **G** - Viability of cells treated with TAT-BID and TRAIL (100 ng/ml) for 24 h, measured by MTT test. The average of cells viability (±SD) was shown. **B, C** – LNCaP. There is no statistically significant difference between viability of TAIL- and TAT-BID-treated LNCaP cells. **D**, **E** – A549. **F**, **G** – HeLa.

The next question we liked to answer was whether TAT-BID sensitized other cancer cell lines to TRAIL similarly as it was observed for PC3. The prostate cancer cell line LNCaP is TRAIL resistant [51]. However, another TRAIL-resistant non-small human lung cancer A549 cell line [52] has been shown to get TRAIL sensitivity upon expression of BID from pDNA vector [8, 11]. Therefore, we tested ability of TAT-BID to sensitize both initially insensitive cell lines to TRAIL. We also used TRAIL-sensitive cervix carcinoma HeLa cells [52]. Estimated levels of endogenous BID in examined cancer cell lines varied from about 10 fg/cell for LNCaP to about 35 fg/cell for A549 cells (Figure 6A).

Although TAT-BID entered into all examined cells, the taken amount was different for particular cell line and did not depend simply on the level of endogenous BID. The amount of TAT-BID that entered cells was the highest in LNCaP cells, moderate in A549 and low in HeLa cells. When the amount of TAT-BID was related to the level of endogenous BID, a standard treatment with TAT-BID (30 or 40 μg/ml for 120 min) resulted in increase of the amount of cellular BID by 100% for LNCaP, 60% for A549 and 20% for HeLa cells (Figure 6B, D, F). Taking into account levels of endogenous BID in the cell lines (Figure 6A), one could calculate that after TAT-BID treatment total amount of BID was within the range 20 – 25 fg/cell for LNCaP and HeLa cells, and about 60 fg/cell for A549 cells.

Similarly as in the case of PC3 cells, TAT-BID did not influence viability of remaining cell lines by more than 10%, at least up to the dose of 40 μg/ml (Additional file 2: Figure S2). TRAIL administered alone at the dose of 100 ng/ml affected viability of neither A549 nor LNCaP cells by more than 5% (Additional file 1: Figure S1B, C). In the latter case, no significant changes in cell viability were observed at concentration as high as 1000 ng/ml. On the contrary, at the TRAIL dose of 100 ng/ml TRAIL alone decreased HeLa cells viability by about 40 - 50% (Additional file 1: Figure S1D).

To compare effects of TAT-BID on different cell lines, we generally used the dose of 100 ng/ml TRAIL (Figure 6C, E, G). At this dose the effect of TAT-BID on sensitivity of LNCaP was not statistically significant (Figure 6C). Neither was it observed at higher TRAIL doses, up to 500 ng/ml (not shown). A549 cells were sensitized by TAT-BID to TRAIL similarly as PC3 cells (Figure 6E). HeLa cells were only slightly sensitized to TRAIL (Figure 6G). Increase of concentration of TAT-BID in the medium up to 50 μg/ml did not change a picture presented on Figure 6C, E, G (not shown). Similarly as it was observed for PC3 cells, unphosphorylable mutant TAT-BID^T59A/S76A^ sensitizes neither A549 nor HeLa cells to TRAIL better than the wild type TAT-BID. It was also ineffective towards LNCaP cells (not shown). None cell line other than PC3 was sensitized by TAT-BID to CPT.

## Discussion

The main finding of this work is that direct delivery of recombinant BID fused with the TAT cell penetrating peptide allows for controlled sensitization of cancer cells to apoptosis induced by TRAIL and CPT. Its results are primarily relevant to potential use of recombinant BID directly delivered to cells in anticancer therapy; however, the work clarifies also some points concerning functioning of BID in different cancer cells.

Importance of BID for apoptosis has been reported for several cancer cell lines that became either more sensitive to apoptosis after expression of BID or more resistant to it after silencing expression of the protein [8–11]. Those reports raised also a question of therapeutic potential of the overexpressed BID that would act alone [8, 10] or combined with chemotherapy for cancer cells [11]. However, to confine effects of extra BID only to cancer cells it has to be applied in low amounts, not enough to kill the cells but rather to sensitize them to agents that induce apoptosis. The system described in this paper fulfills this condition because direct delivery of TAT-BID allows for stringent control of the amount of BID in particular type of cancer cells. As an alternative to expression from DNA vectors, cell penetrating peptides offer fast, efficient and dose-dependent transport of proteins required for their therapeutic use [31]. Such characteristics was observed in this work for TAT-BID uptake (Figures 2 and 6) and thus pointed to the potential of BID fused with TAT as a therapeutic strategy.

The results presented here show that TAT-BID sensitizes PC3, A549 and to very low extent HeLa cells to apoptosis induced by TRAIL, but it fails to sensitize LNCaP cells. Looking for explanation of this picture we firstly analyzed transportation of TAT-BID into cells. Different cell lines do not show the same efficiency of the uptake of cell penetrating peptides conjugates [53]. It was also observed by us that transportation occurred with different efficacy in particular cell lines: it was high for LNCaP cells, slightly lower for PC3 and A549 cells and very low for HeLa cells. In the latter case poor transportation of TAT-BID correlated with low detectable sensitization of HeLa cells to TRAIL although a cause and effect relationship between the two phenomena was not proved here. However, transportation into cancer cells has to be considered as a factor that could influence therapeutic use of TAT-BID. Another question to be answered was why TAT-BID overcame TRAIL resistance of A549 but not LNCaP cells, despite of satisfactory transportation of TAT-BID into cells of both cell lines. As concerns the first cell line, this is in agreement with previous findings that expression of BID from pDNA expression vector sensitizes A549 cell to apoptosis induced by TRAIL [8, 11]. It seems probable that different effects of TAT-BID on LNCaP and A549 result from different sites at which defects in TRAIL-induced signaling are localized. In LNCaP cells the signaling is impaired upstream of BID at the stage of c-FLIP [51, 54], whereas in A549 cells downstream of BID at the stage of Bcl-2 that is overexpressed in these cells [55, 56]. In the latter case total amount of cellular BID necessary to make A549 cells sensitive to TRAIL was significantly higher than that effective in PC3 cells (Figures 2 and 6). Since Bcl-2 sequesters active tBID and this way prevents cells from killing [19], it is probable that more BID is necessary in A549 than in PC3 cells to start apoptosis. The above observation also indicates that estimation of a level of endogenous BID cannot be a prediction of sensitivity of cancer cells to TAT-BID.

We showed here that TAT-BID sensitized PC3 cells to apoptosis induced by DNA damage introduced by CPT (Figure 4D) although sensitization to CPT was clearly weaker than that to TRAIL. The question was why PC3 but not any other cell line examined in this work was sensitized to CPT by excess of BID. The pronounced determinant of PC3 making them distinct from other cells used here is lack of active p53 [57] that mediates main cellular response to DNA damage. It might be thus thought that p53-indepentent apoptosis following CPT-induced DNA damage in PC3 cells was more prone to BID level than that activated in cells containing active p53. A functional connection between BID and p53 has been shown in HCC cells [17]. It has also been shown that cells of HCC line bearing mutated inactive p53 are sensitized by overexpressed BID to DNA damage introduced by etoposide [28] to the extent similar to that observed here for PC3 cells sensitized by TAT-BID to CPT.

Although direct comparison of CK2 activity in all four cell lines studied here has not been done, several reports provide evidence for elevated activity in each case [22, 24, 58]. However, our results indicated that CK2-mediated phosphorylation neither influenced efficacy of TAT-BID delivered to PC3 and A549 nor contributed to lack of effect of TAT-BID on LNCaP and to poor sensitization of HeLa cells. Therefore, sensitization of cancer cells to apoptosis based on substitution of BID by its unphosphorylable analogue does not seem to be better strategy at least for cancer lines studied in this work.

TAT is not specific to cancer cells and additional mechanisms have to be employed to restrict sensitization made by TAT-BID to tumors. An obvious solution is a combined treatment including TAT-BID and an anticancer drug that is cancer cells-specific. This is a case of TRAIL that induces apoptosis in cancer but not in normal cells [15], and that has been shown here as effective against PC3 and A549 cells if combined with TAT-BID. Another possibility is to fuse TAT-BID with additional cassette that restricts its action to tumor cells. Such a cassette is ODD domain [59] that accelerates degradation of the fusion protein under normoxia, whereas the fusion protein remains stable under hypoxia conditions, i.e. inside tumors. Results of our ongoing investigations indicate that TAT-BID collaborate well with ODD cassette. These preliminary results show that enrichment of TAT-BID in additional targeting cassette is a promising way to improve its specificity to cancer cells.

## Conclusions

In summary, our results indicate that recombinant BID fused with TAT peptide may be delivered to cells in controlled manner so that it is not toxic but it sensitizes cancer cells to apoptosis. The results point to possible therapeutic potential of BID fused with TAT for cancer treatment. If applied to cell cultures, controlled delivery of BID allows for simple examination of sensitivity of different cancer cells to BID-based therapies and of efficacy of different agents in the treatment combined with BID. The findings presented here indicate that PC3 and A549 cells are good targets for TAT-BIB, and that TRAIL is particularly effective if combined with TAT-BID.

## Electronic supplementary material

Additional file 1: Figure S1: Effect of TRAIL on viability of the used cell lines. Viability of cells was measured by MTT test. The average of cells viability (±SD) is shown. Red – 100 ng/ml; blue – 500 ng/ml; black – 1000 ng/ml. (TIFF 2 MB)

Additional file 2: Figure S2: Effect of TAT-BID on viability of LNCaP, A549 and HeLa cells. Viability of cells treated with TAT-BID for 24 h was measured by MTT test. The average of cells viability (±SD) is shown. Pink – LNCaP; yellow – A549; green – HeLa cells. (TIFF 281 KB)

## Abbreviations

BID: BH3-interacting domain death agonist
CPT: Camptothecin
TAT-BID: Recombinant BID fused with TAT cell penetrating peptide
tBID: Truncated form of BID
TRAIL: Recombinant soluble TNF-related apoptosis inducing ligand.
