# Supplementary figures and images for: Controlled delivery of BID protein fused with TAT peptide sensitizes cancer cells to apoptosis
Source: BMC Cancer. 2014 Oct 18;14:771. doi: 10.1186/1471-2407-14-771 (PMC4210496; doi:10.1186/1471-2407-14-771)

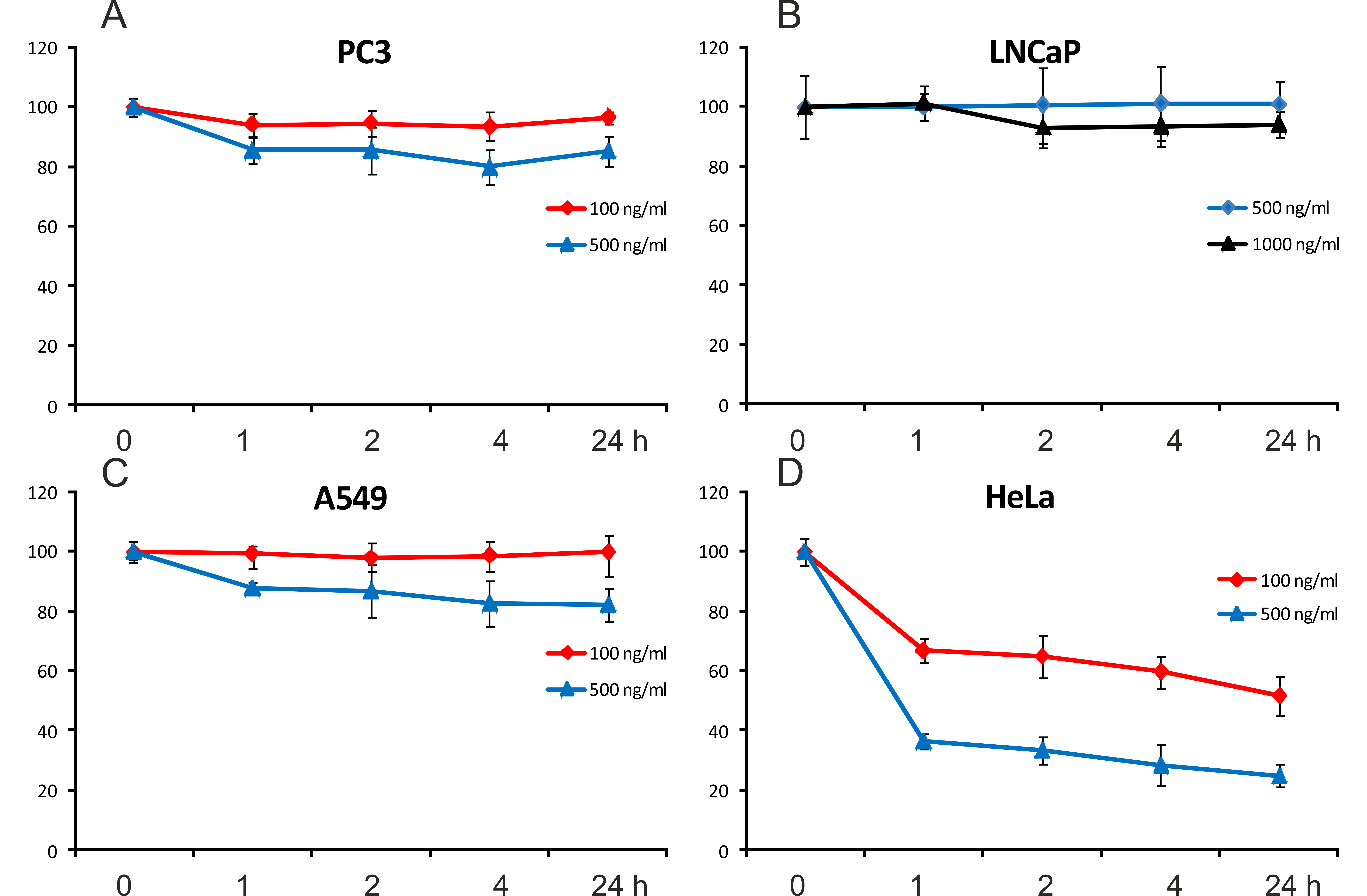

Supplement: Supplementary file 1 — Additional file 1: Figure S1: Effect of TRAIL on viability of the used cell lines. Viability of cells was measured by MTT test. The average of cells viability (±SD) is shown. Red – 100 ng/ml; blue – 500 ng/ml; black – 1000 ng/ml. (TIFF 2 MB) [file 12885_2014_4949_MOESM1_ESM.tiff]

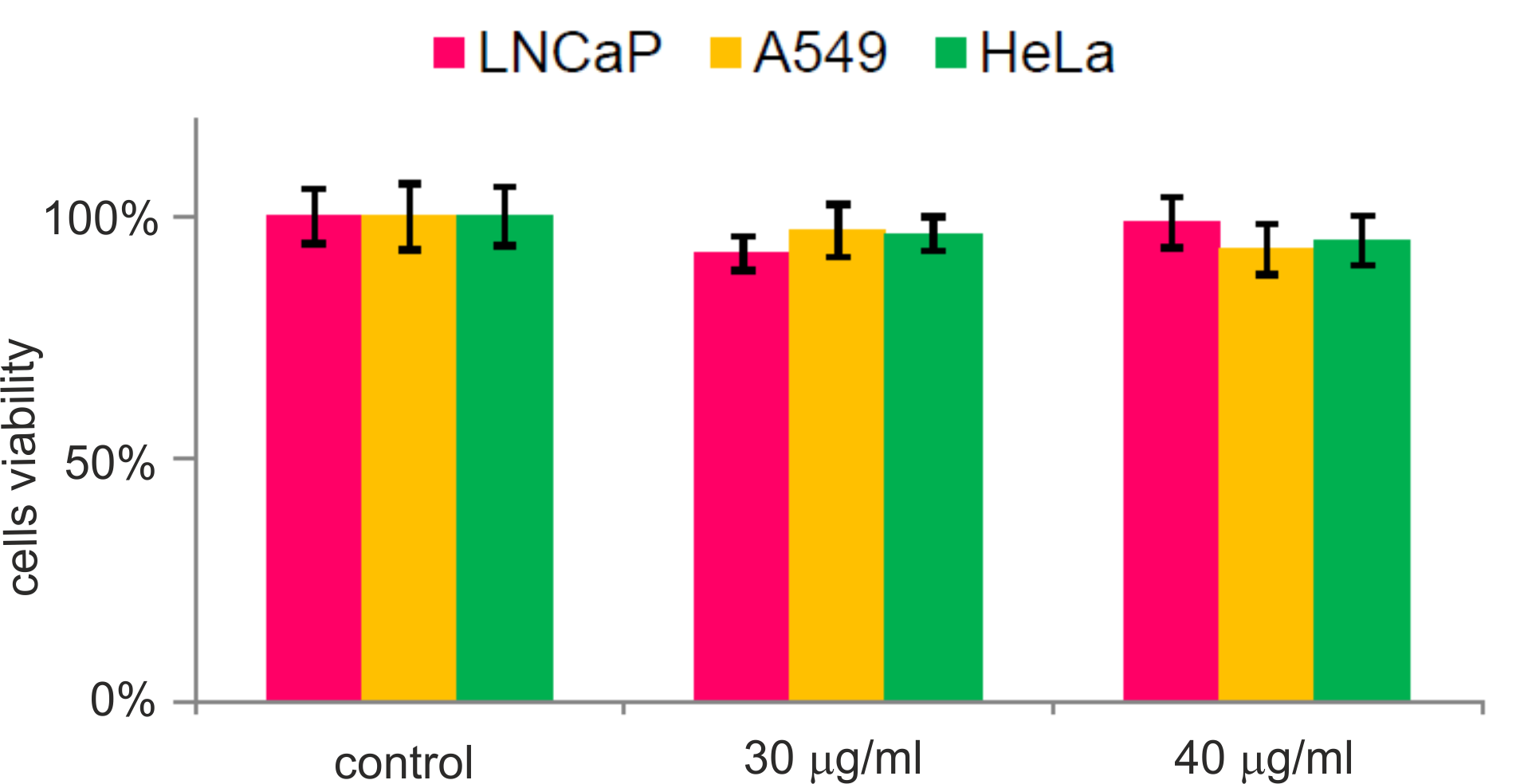

Supplement: Supplementary file 2 — Additional file 2: Figure S2: Effect of TAT-BID on viability of LNCaP, A549 and HeLa cells. Viability of cells treated with TAT-BID for 24 h was measured by MTT test. The average of cells viability (±SD) is shown. Pink – LNCaP; yellow – A549; green – HeLa cells. (TIFF 281 KB) [file 12885_2014_4949_MOESM2_ESM.tiff]
